# Inhibiting checkpoint kinase 1 protects bone from bone resorption by mammary tumor in a mouse model

**DOI:** 10.18632/oncotarget.24286

**Published:** 2018-01-19

**Authors:** Shengzhi Liu, Yang Liu, Kazumasa Minami, Andy Chen, Qiaoqiao Wan, Yukun Yin, Liangying Gan, Aihua Xu, Nariaki Matsuura, Masahiko Koizumi, Yunlong Liu, Sungsoo Na, Jiliang Li, Harikrishna Nakshatri, Bai-Yan Li, Hiroki Yokota

**Affiliations:** ^1^ Department of Pharmacology, School of Pharmacy, Harbin Medical University, Harbin 150081, China; ^2^ Department of Biomedical Engineering, Indiana University at Purdue University, Indianapolis, IN 46202, USA; ^3^ Department of Medical Physics and Engineering, Osaka University Graduate School of Medicine Suita, Osaka 565-0871, Japan; ^4^ Department of Biology, Indiana University at Purdue University, Indianapolis, IN 46202, USA; ^5^ Osaka Medical Center for Cancer and Cardiovascular Diseases, Osaka 537-8511, Japan; ^6^ Department of Medical and Molecular Genetics, Indiana University School of Medicine, Indianapolis, IN 46202, USA; ^7^ Department of Surgery, Simon Cancer Research Center, Indiana University School of Medicine, Indianapolis, IN 46202, USA

**Keywords:** breast cancer, bone resorption, checkpoint kinase, eIF2α

## Abstract

DNA damage response plays a critical role in tumor growth, but little is known about its potential role in bone metabolism. We employed selective inhibitors of Chk1 and examined their effects on the proliferation and migration of mammary tumor cells as well as the development of osteoblasts and osteoclasts. Further, using a mouse model of bone metastasis we evaluated the effects of Chk1 inhibitors on bone quality. Chk1 inhibitors blocked the proliferation, survival, and migration of tumor cells *in vitro* and suppressed the development of bone-resorbing osteoclasts by downregulating NFATc1. In the mouse model, Chk1 inhibitor reduced osteolytic lesions and prevented mechanical weakening of the femur and tibia. Analysis of RNA-seq expression data indicated that the observed effects were mediated through the regulation of eukaryotic translation initiation factor 2 alpha, stress to the endoplasmic reticulum, S100 proteins, and bone remodeling-linked genes. Our findings suggest that targeting Chk1 signaling without adding DNA damaging agents may protect bone from degradation while suppressing tumor growth and migration.

## INTRODUCTION

The genome is constantly exposed to DNA damaging agents, including radiation and chemical mutagens [1]. Eukaryotic cells have evolved signaling pathways that detect these insults and protect them from DNA damage [2]. One of the primary pathways to respond to DNA damage is checkpoint kinases 1 and 2 (Chk1 and Chk2) signaling [3]. Activation of Chk1 and Chk2 results in cell cycle arrest to elicit DNA repair or apoptosis [4]. Mutations to these Chk genes are reported to be linked to cancers, including breast cancer [5, 6]. Since Chk1 and Chk2 are essential molecular players in responding to DNA damaging agents, application of their inhibitors together with DNA damaging chemotherapeutic drugs has been considered and clinically tested [7]. To our knowledge, however, little is known about the role of Chk signaling in modulating the activity of bone-resorbing osteoclasts and bone-forming osteoblasts in response to cancer metastasis.

Breast cancer accounts for 25% of all cancers in women, and advanced breast cancer metastasizes to distant organs, most commonly bone [8]. Thus, developing an effective therapeutic agent for reducing metastatic burden and protecting bone quality without inducing life-threatening toxicities is necessary [9]. We screened novel drug candidates from a 1,120-compound chemical library using two breast cancer cell lines (MDA-MB-231 and MDA-MB-436) and two non-tumor cell lines. We selected a Chk1 inhibitor (PD407824) as well as a dopamine receptor D1 agonist (A77636), which reduced the proliferation of two cancer cell lines without significant reduction of proliferation of two non-tumor cell lines. The efficacy of A77636 in suppressing tumor growth and bone loss was reported previously [10].

It was recently reported that the inhibition of Chk1 by PF477736 elevates cytotoxicity and sensitizes embryonic Ras-transformed fibroblast cells in response to reduction in nucleotide pools [11]. To further evaluate the therapeutic potential of Chk1 inhibitors for mammary tumor and associated bone metastasis, we examined their ability to inhibit proliferation and migration of mammary tumor cells and to prevent bone loss. RNA sequencing was employed to evaluate genome-wide expression changes. Expression patterns were characterized by principal component analysis (PCA). To test the effects of Chk1 inhibitor *in vivo*, 4T1.2 mammary tumor cancer cells were injected into the mammary pad or the external iliac artery.

We examined the mechanism of Chk1 inhibitor’s action focusing on phosphorylation of eukaryotic translation initiation factor 2 alpha (eIF2α), stress to the endoplasmic reticulum, S100 proteins, bone remodeling-linked genes, and Ataxia telangiectasia and Rad3 related (ATR) signaling. We have previously shown that upregulation of the phosphorylation of eIF2α by salubrinal and guanabenz decreases proliferation of mammary tumor cells and chondrosarcoma cells [12, 13]. We have also shown that the elevated phosphorylation of eIF2α downregulates nuclear factor of activated T cells (NFATc1), a master transcription factor for development of bone-resorbing osteoclasts [14, 15]. We applied RNA interference and targeted chemical inhibitors to elucidate the signaling relationships between these pathways and Chk1 inhibition.

## RESULTS

### Chk1 inhibition suppresses tumor growth and migration

**Figure 1:**
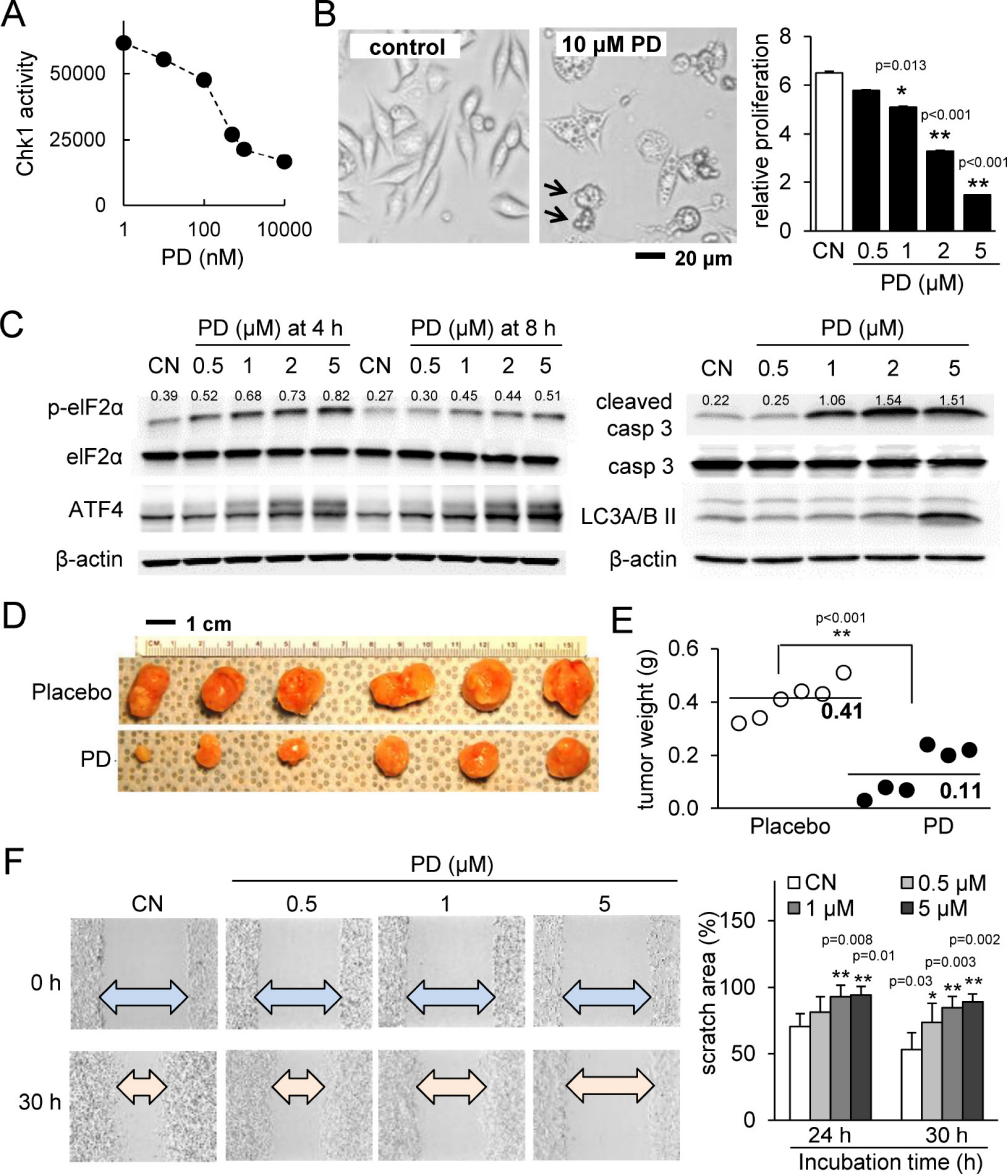
PD407824 suppresses tumor growth and migration CN *=* control. (**A**) Chk1 activity is inhibited by increasing dosages of PD407824. (**B**) Representative cell images in response to 10 μM PD407824 for 24 h, and relative proliferation of 4T1.2 mammary tumor cells in response to 0.5, 1, 2, and 5 μM PD407824 for 3 days. The arrows indicate cells undergoing apoptosis. (**C**) Elevation of p-eIF2α and ATF4 protein in response to 0.5-5 μM PD407824 for 4 and 8 h, as well as elevation of cleaved caspase 3 (apoptosis marker) and LC3A/B II (autophagy marker) for 24 h. (**D–E**) Reduction of tumor size by daily intraperitoneal injection of PD407824 at 2 mg/kg for 3 weeks (*N* = 6). Mice received injection of 4T1.2 cells (5.0 × 10^5^ cells in 50 μl PBS) at the mammary fat pad. (**F**) Dose-dependent reduction in cell motility of 4T1.2 cells in response to PD407824 in a scratch assay for 24 h and 30 h.

PD407824 was found to inhibit Chk1 activity in a dose-dependent manner (Figure 1A). Treatment of 4T1.2 mammary tumor cells with PD407824 induced apoptosis and autophagy and reduced cellular proliferation (Figure 1B). We have previously shown that a modulator of p-eIF2α, salubrinal, induced apoptosis to 4T1 cells [14]. PD407824 also elevated p-eIF2α, as well as cleaved caspase 3 (apoptosis marker) and LC3A/B II (autophagy marker) (Figure 1C). Treatment of MDA-MB-231 breast cancer cells showed similar effects (Supplementary Figure 1), while non-tumorigenic epithelial cells CRL-3063 are less sensitive to both PD407824 and PF477736 than 4T1.2 cells (Supplementary Figure 5). Two clones of MD-MB-231 cells (tumor-derived TMD and bone metastasis-derived BMD) as well as three mouse mammary tumor cell lines were also found to have decreased proliferation from PD407824 treatment (Supplementary Figure 1). Injection of 4T1.2 cells into the mammary fat pad induced a solid tumor at the injection site with an average weight of 0.41 g (*N* = 6) (Figure 1D and 1E). Daily intraperitoneal injection of PD407824 at 2 mg/kg significantly reduced tumor weight (0.11 g on average; *N* = 6). In a scratch assay, 0.5 through 5 μM PD407824 significantly decreased motility in a concentration-dependent manner (Figure 1F).

### Chk1 inhibition reduces osteoclastogenesis in RAW264.7 pre-osteoclasts

**Figure 2:**
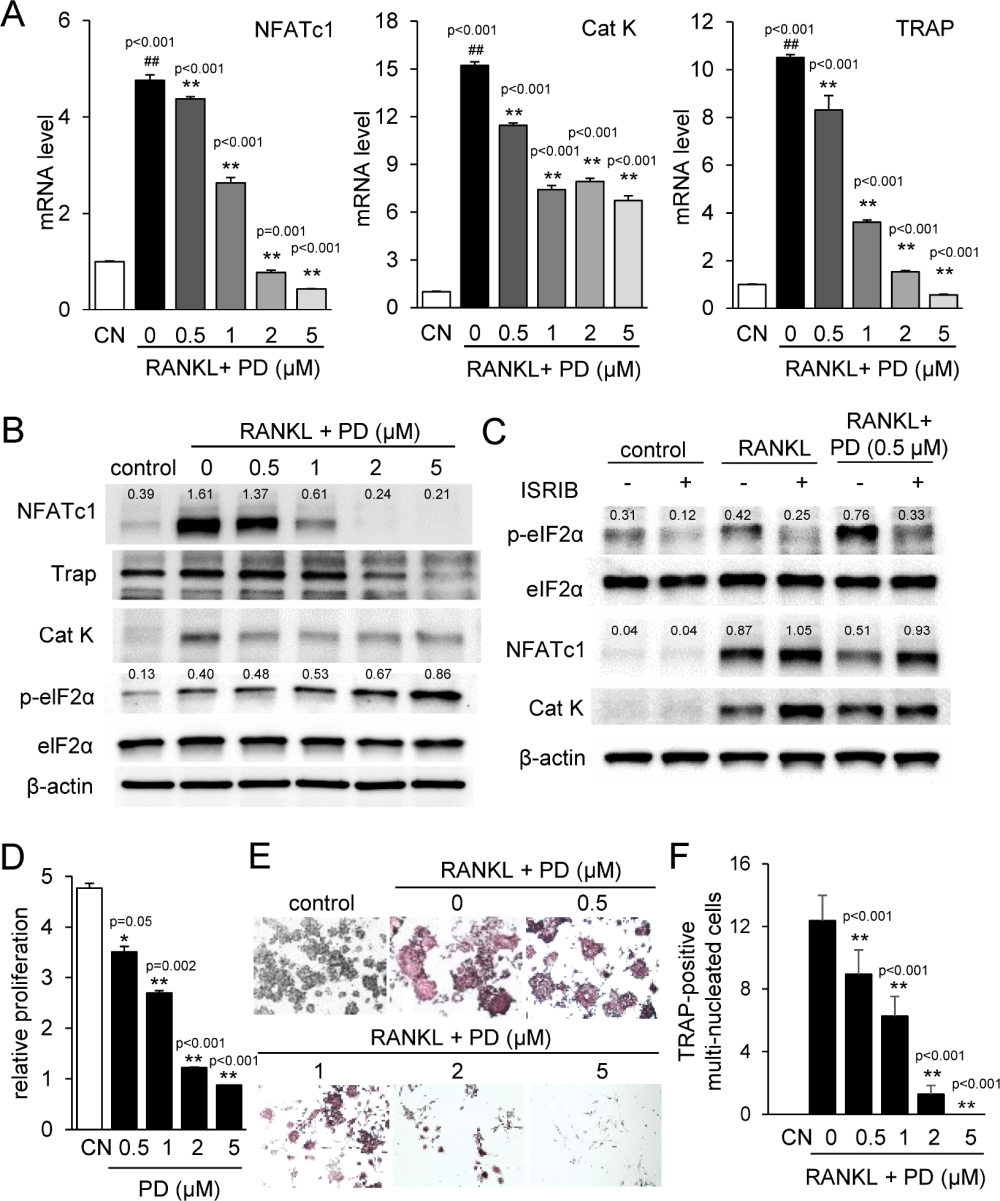
PD407824 inhibits osteoclastogenesis in RAW264.7 pre-osteoclasts Data are represented as the means ± SD of three independent experiments, in which ^##^ and ^**^ indicate *p* < 0.01 compared to the control and RANKL groups, respectively. CN *=* control. (**A**) Reduction of the mRNA levels of NFATc1, Cathepsin K (Cat K), and TRAP in RANKL-stimulated RAW264.7 cells in response to 0.5–5 μM PD407824 in 24 h. (**B**) Reduction of the protein levels of NFATc1, TRAP, and Cat K, as well as elevation of the phosphorylation level of eIF2α (p-eIF2α) by 0.5–5 μM PD407824 in 24 h. (**C**) Effects of 10 μM ISRIB, an inhibitor of eIF2α phosphorylation, on the protein levels of NFATc1 and Cat K. (**D**) Dose-dependent reduction in relative proliferation of RAW264.7 cells by 0.5 to 5 μM PD407824 for 3 days. (**E**) Inhibition of TRAP-positive mature osteoclasts by RANKL-stimulated RAW264.7 cells from 0.5–5 μM PD407824 after 4 days. (**F**) Number of TRAP-positive multi-nucleated cells in RANKL-treated RAW264.7 cells.

We next examined the effects of PD407824 on osteoclasts. In response to PD407824, the mRNA and protein levels of NFATc1, Cathepsin K, and TRAP were downregulated (Figure 2A and 2B). As in 4T1.2 cells, p-eIF2α was also upregulated (Figure 2B). In response to ISRIB, an inhibitor of eIF2α phosphorylation, the PD407824-mediated reduction in NFATc1 and Cat K was suppressed (Figure 2C). Furthermore, proliferation of RAW264.7 pre-osteoclasts was reduced by 0.5 to 5 μM PD407824 (Figure 2D). TRAP staining revealed that induction of TRAP-positive osteoclasts was strongly suppressed by 1–5 μM PD407824 (Figure 2E and 2F). A set of *in vitro* experiments conducted using PF477736, another selective Chk1 inhibitor, demonstrated similar results to PD407824 in osteoclast differentiation (Supplementary Figure 2) as well as suppression of NFATc1 and Cat K expression (Supplementary Figure 4). AZ20, an inhibitor of Chk1-stimulating ATR signaling, also suppresses NFATc1 and Cat K expression (Supplementary Figure 4).

### Principal component analysis (PCA) of gene expression changes by Chk1 inhibition

**Figure 3:**
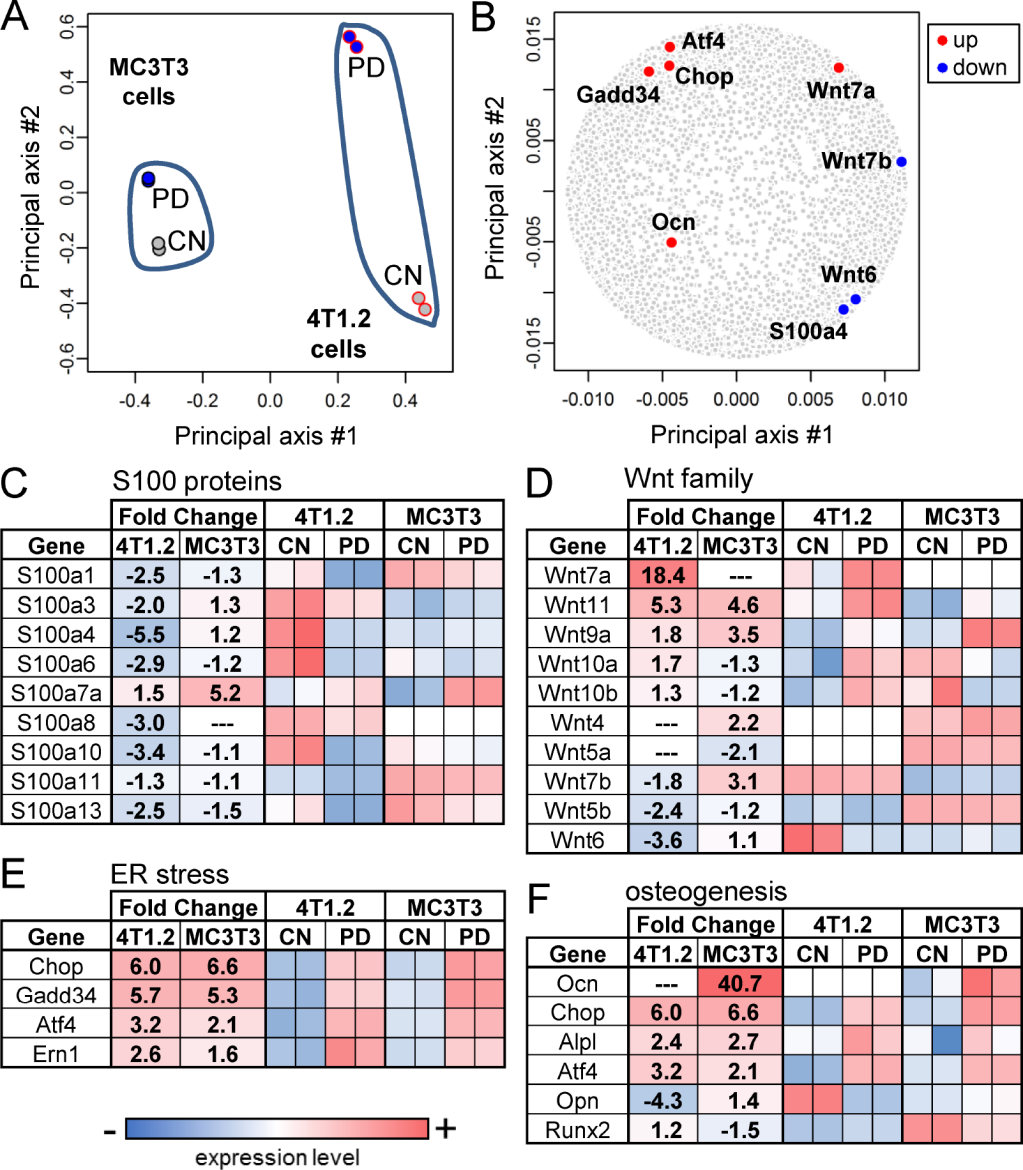
PCA and genome-wide gene expression profiles CN *=* control. (**A**) Locations of 4 samples in the principal component plane. Two samples from MC3T3 cells are located closer each other than two samples from 4T1.2 cells. (**B**) Locations of the selected PD407824-sensitive genes in the principal component plane. (**C**) Alteration in mRNA levels of S100 calcium-binding proteins (S100 proteins). (**D–F**) Alterations in mRNA levels of the genes related to Wnt signaling, the stress to the endoplasmic reticulum (ER), and osteogenesis, respectively.

In response to PD407824, we determined genome-wide mRNA expression profiles of 13,920 genes in 4T1.2 cells and MC3T3 osteoblast cells and conducted PCA (Figure 3A). The first principal axis primarily corresponded with the differential expression between MC3T3 and 4T1.2 cells, while the second principal axis aligned with the effect of PD407824. The selected genes, which were located in the principal plane (Figure 3B), were highly responsive to PD407824 and related to 4 pathways: S100 calcium-binding protein linked pathway (S100a4), Wnt ligands (Wnt6, Wnt7a, and Wnt7b), stress to the endoplasmic reticulum (ATF4 and GADD34), and osteogenesis (ATF4 and osteocalcin - OCN) (Figure 3C–3F).

**Figure 4:**
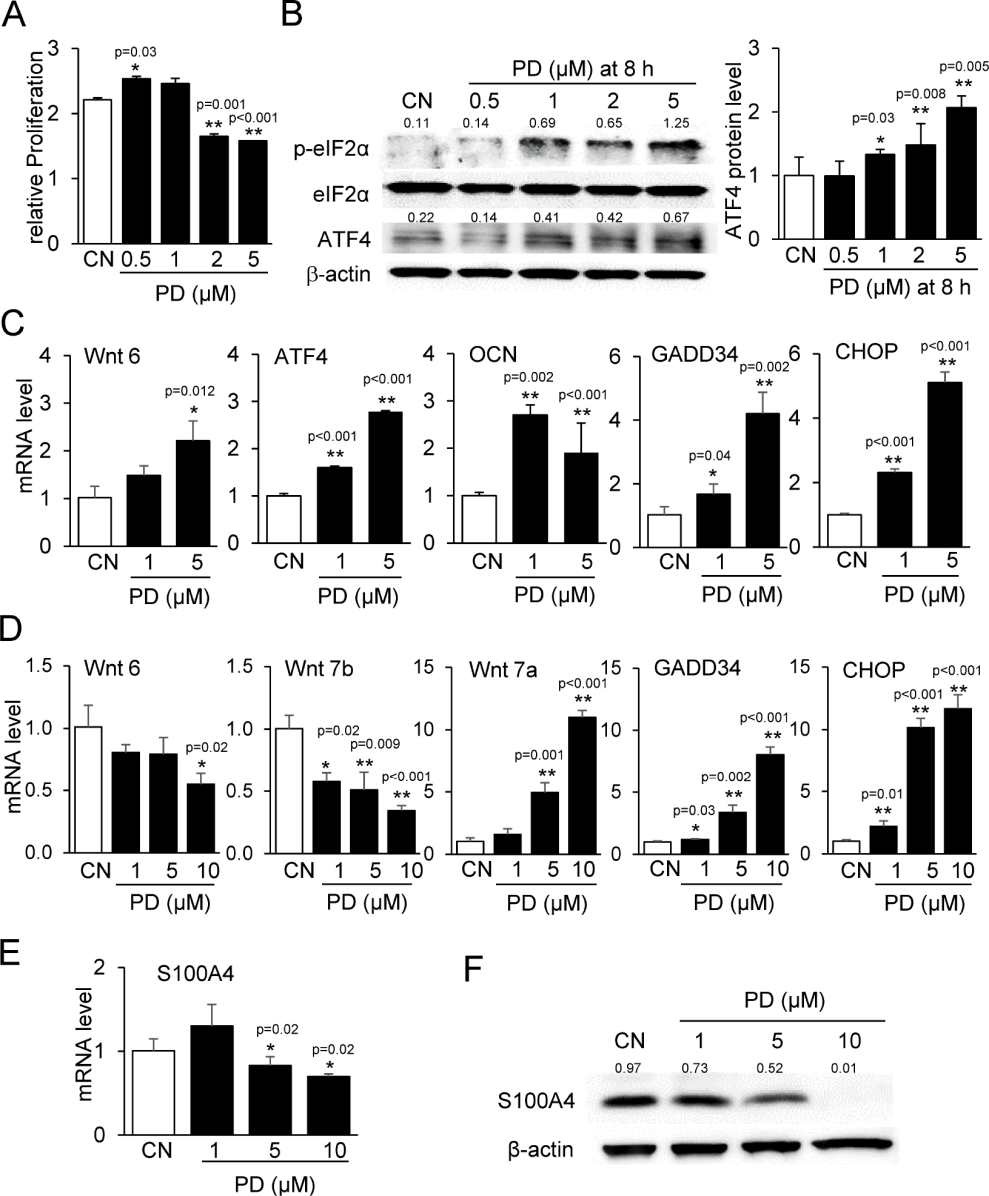
Validating RNA-seq-derived pathways in MC3T3 and 4T1.2 cells CN = control. (**A**) Alteration in proliferation of MC3T3 cells by 0.5–5 μM PD407824 on day 3. (**B**) Increase in the protein levels of p-eIF2α and ATF4 in response to 0.5–5 μM PD407824 at 8 h in MC3T3 cells. (**C**) Increase in the mRNA levels of Wnt6, ATF4, osteocalcin (OCN), GADD34, and CHOP in response to 1 and 5 μM PD407824 in MC3T3 cells. (**D**) Alterations in the mRNA levels of Wnt6, Wnt7b, Wnt7a, GADD34, and CHOP in 4T1.2 cells. (**E**) Reduction of S100A4 mRNA level by 5 and 10 μM PD407824 in 4T1.2 cells. (**F**) Reduction of the protein level of S100A4 by PD407824 in 4T1.2 cells.

Using qPCR, we confirmed some of the RNA-seq-derived genes of interest. PD407824 at lower concentrations (0.5 and 1 μM) elevated proliferation of MC3T3 cells, while at higher concentrations (2 and 5 μM) it reduced their proliferation (Figure 4A). PD407824 elevated Wnt6, ATF4, OCN, GADD34, and CHOP mRNAs in MC3T3 cells and increased p-eIF2α and ATF4 protein (Figure 4B and 4C). In 4T1.2 cells, PD407824 reduced the mRNA levels of Wnt6 and Wnt7b and elevated Wnt7a, GADD34, and CHOP mRNAs (Figure 4D). In 4T1.2 cells, PD407824 reduced the mRNA and protein levels of S100A4, S100 calcium-binding protein A4 (Figure 4E and 4F). PF477736 exhibited similar effects as PD407824 on MC3T3 cell proliferation, p-eIF2α, ATF4 expression, and MC3T3 mineralization (Supplementary Figures 3 and 4).

### Chk1 inhibition protects bone strength and structure from bone metastasis

In the mouse model of bone metastasis, 4T1.2 cells expressing GFP were injected. On days 1 to 3 after tumor inoculation, we conducted qPCR for GFP to examine the presence of tumor cells in the bone and bone marrow. The results showed that the GFP DNA level was detectable on day 1 and significantly increased on day 3, indicating that the colonization was initiated on day 1 (Supplementary Figure 4). On day 18 after inoculation of 4T1.2 cells, animals were sacrificed. During this experiment, administration of PD407824 did not significantly change body weight (Supplementary Figure 4).

**Figure 5:**
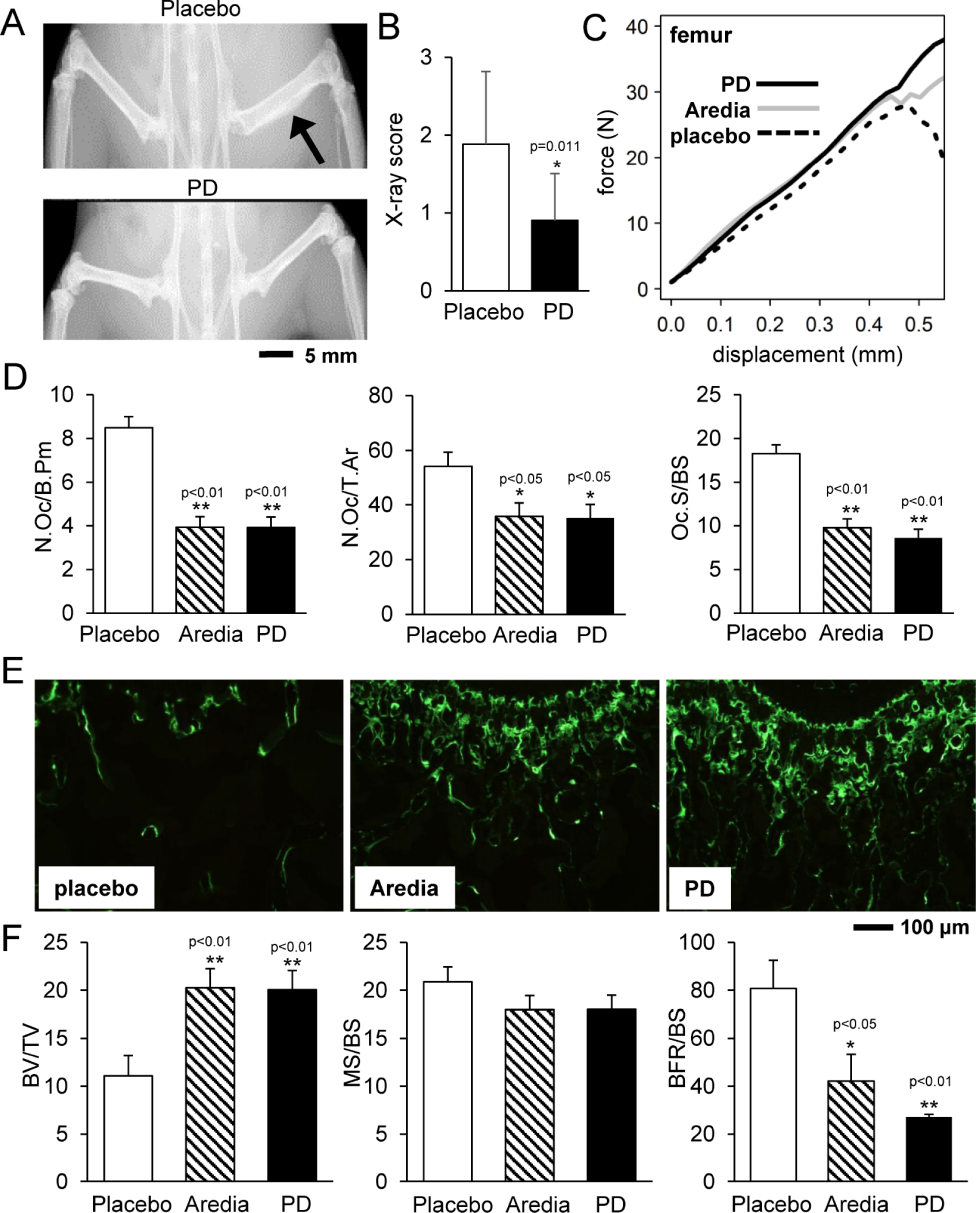
PD407824 protects bone strength and structure in the mouse model of bone metastasis (**A** and **B**) Representative X-ray images of the hindlimb of the placebo and PD407824-treated mice, and X-ray score of the femur (*N* = 12) in the scale of 0 (normal) to 3 (severe morphological change). (**C**) Average curve for the force-displacement relationship of the femur in the four-point mechanical test (*N* = 12). (**D**) Characteristic parameters of trabecular bone in the distal femur in TRAP-stained sections (*N* = 12). Of note, N.Oc/B.Pm = number of osteoclasts normalized by bone perimeter; N.Oc/T.Ar = number of osteoclasts normalized by tissue area; and Oc.S/BS = osteoclast surface normalized by bone surface. (**E** and **F**) Representative images of calcein-stained sections of trabecular bone in the distal femur, and three parameters for bone formation. BV/TV = bone volume normalized by tissue volume; MS/BS = mineralizing surface normalized by bone surface; and BFR/BS = bone formation rate normalized by bone surface.

X-ray imaging revealed that, compared to the placebo group, PD407824-treated group exhibited a milder degree of phenotypical bone deformation (Figure 5A). Visually scoring the X-ray images of the femur (*N* = 12) on a scale of 0 (normal) to 3 (severe morphological change) showed that the placebo and PD407824-treated groups scored on average 1.89 ± 0.93 and 0.90 ± 0.60, respectively (Figure 5B). The force-displacement relationship of the femur (*N* = 12) revealed that groups treated with PD407824 and Aredia (a bisphosphonate that inhibits osteoclast function) had a higher stiffness (i.e., slope) than the placebo group, and the PD407824-treated group presented the highest peak force (Figure 5C; Supplementary Figure 6).

In the TRAP-stained sections of the distal femur, the number of osteoclasts and osteoclast surface were significantly smaller in PD407824- and Aredia-treated groups than that of the placebo group (Figure 5D; Supplementary Figure 6). In the calcein-stained sections, the bone volume was greater in PD407824- and Aredia-treated groups than that of the placebo group (Figure 5E). However, the bone formation rate normalized by bone surface (BFR/BS) was the highest in the placebo group due to its smaller bone surface (Figure 5F).

### Histological and μCT analysis of the distal femur in response to Aredia and PD407824

**Figure 6:**
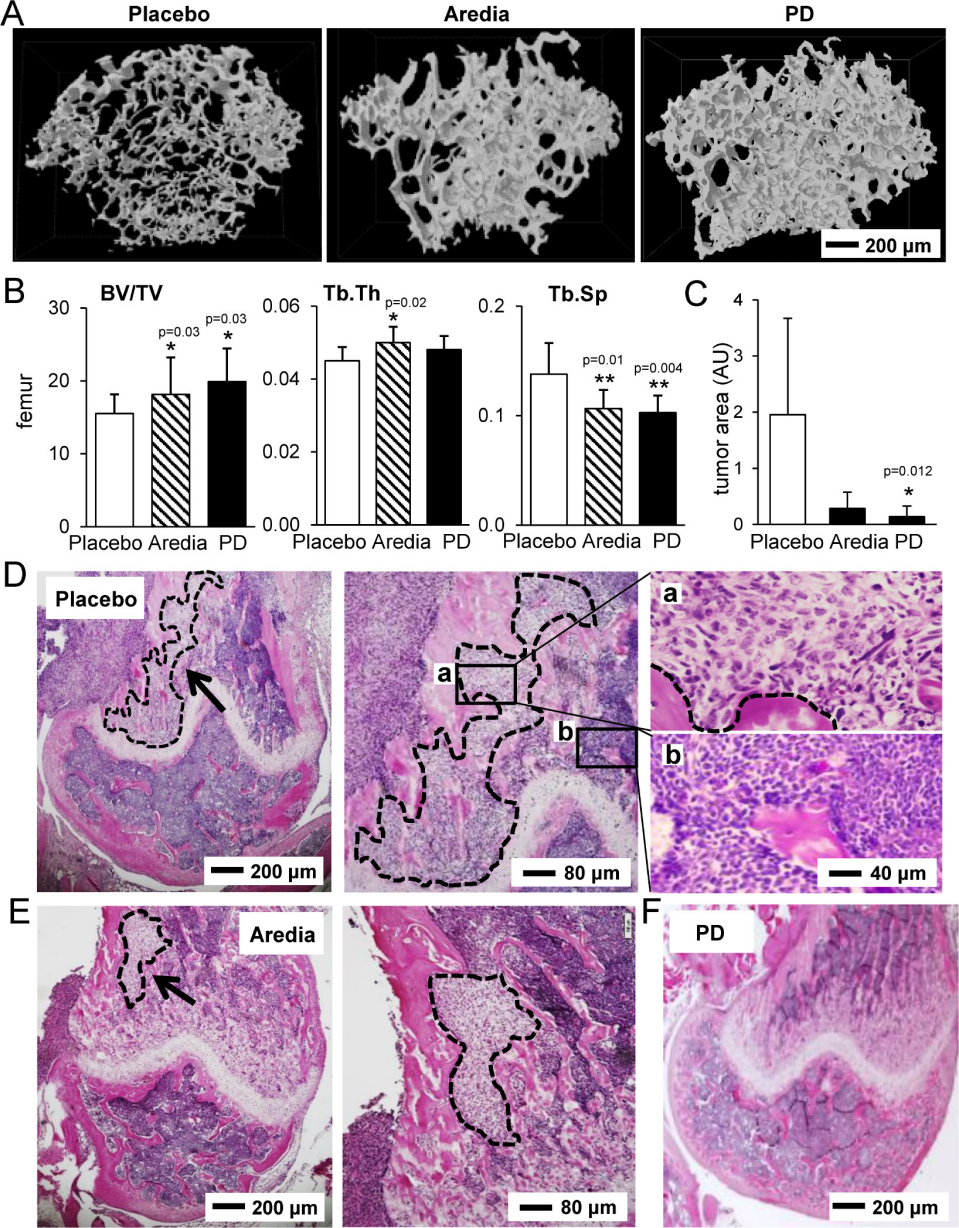
Histological and μCT analysis of the distal femur in response to Aredia and PD407824 (**A**) Representative μCT images of three groups (placebo, Aredia, and PD407824). (**B**) Three parameters for trabecular bone in the distal femur. Of note, BV/TV = bone volume normalized by tissue volume; Tb.Th = trabecular thickness; and Tb.Sp = trabecular separation. (**C**) Comparison of the invaded tumor area in the sagittal sections of the distal femur. (**D–F**) Representative H&E stained sagittal sections of the distal femur in the three groups (placebo, Aredia, and PD407824). The area with the dotted line indicates tumor cells.

Consistent with the results from mechanical testing and TRAP/calcein staining, μCT-based reconstruction of the distal femur revealed that bone volume was highest in the PD407824-treated group, while trabecular spacing was smaller in Aredia- and PD407824-treated groups than in the placebo group (Figure 6A and 6B). H&E stained sagittal sections revealed that the PD407824-treated group significantly reduced the invasion of tumor cells in the distal femur (Figure 6C–6F).

### Chk1 inhibition acts on p-eIF2α

**Figure 7:**
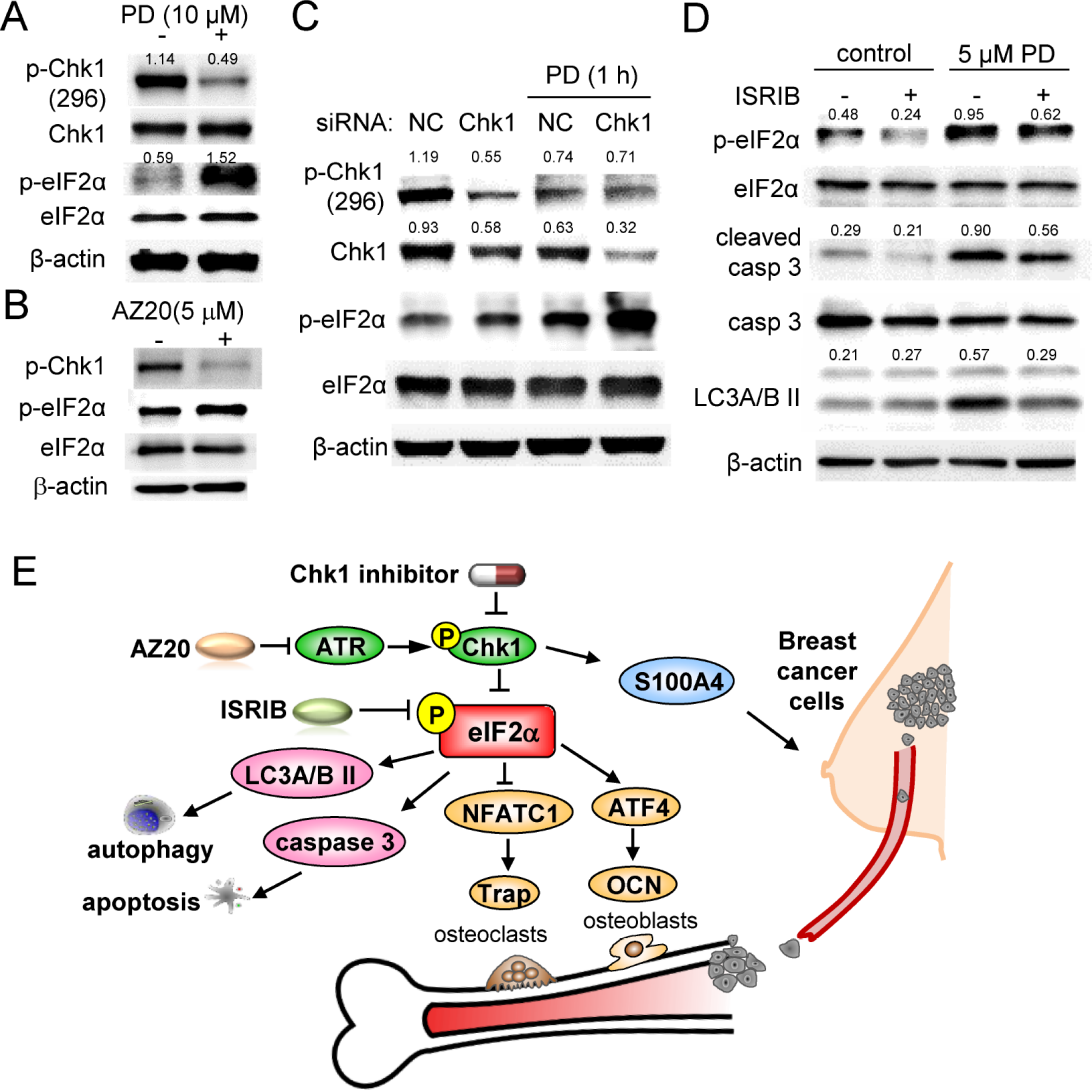
PD407824-driven signaling pathways in 4T1.2 cells Of note, CN = control, and NC = non-specific siRNA control. (**A**) PD407824-driven decrease in p-Chk1. (**B**) Alterations in the protein levels of p-Chk1 and p-eIF2α in response to AZ20. (**C**) Increase in p-eIF2α in response to a partial silencing of Chk1 by RNA interference. (**D**) Suppression of PD407824-driven increase in cleaved caspase 3 by ISRIB, a pharmacological inhibitor of eIF2α phosphorylation. (**E**) Proposed mechanism of PD407824’s action.

We have demonstrated that p-eIF2α is elevated by two Chk1 inhibitors and an ATR inhibitor (AZ20) in 4T1.2 cells. To further investigate the link between Chk1 and eIF2α, we employed RNA interference. In 4T1.2 mammary tumor cells, 10 μM PD407824 decreased p-Chk1 while it elevated p-eIF2α (Figure 7A). AZ20, an inhibitor of Chk1-stimulating ATR signaling, also decreased p-Chk1 level while increasing p-eIF2α (Figure 7B). In response to a partial silencing of Chk1 by RNA interference, PD407824-induced elevation of p-eIF2α appears to be enhanced (Figure 7C). Moreover, treatment of 4T1.2 cells with 10 μM ISRIB, a pharmacological inhibitor of eIF2α phosphorylation, suppressed PD407824-induced expression of cleaved caspase 3 and LC3A/B II (Figure 7D).

## DISCUSSION

In this study, we examined the overall hypothesis that Chk1 inhibitors such as PD407824 and PF477736 can suppress mammary tumor growth and osteolytic lesions by regulating pathways associated with the ATR-Chk1 axis, eIF2α and stress to the endoplasmic reticulum, as well as bone remodeling-linked genes such as NFATc1, and ATF4. Using agents such as AZ20 (ATR inhibitor) and ISRIB (inhibitor of eIF2α phosphorylation), this study demonstrates that Chk1 inhibitors are able to prevent tumor-induced bone loss by blocking tumor growth as well as bone resorption. *In vitro* results showed that PD407824 and PF477736 were potent inhibitors of proliferation and migration of 4T1.2 tumor cells by upregulating cleaved caspase 3 as well as effective suppressors of NFATc1, the master transcription factor of osteoclastogenesis. Animal experiments revealed that PD407824 significantly reduced tumor size. Furthermore, mechanical testing, μCT imaging, and histological analysis indicated that PD407824 was capable of protecting the structure and strength of the femur and tibia from bone resorption in the mouse model.

One of our primary targets is the regulation of p-eIF2α [16]. We have previously shown that salubrinal is able to prevent tumor growth and bone resorption in mice [14, 17]. Salubrinal is known as an inhibitor of protein phosphatase 1 (PP1), which de-phosphorylates eIF2α [18]. We have also reported that another inhibitor of PP1, guanabenz, inhibits development of bone-resorbing osteoclasts but stimulates development of bone-forming osteoblasts [19]. In this study, Chk1 inhibitors significantly elevated p-eIF2α as effectively as salubrinal or guanabenz. The elevated level of p-eIF2α induced apoptosis and autophagy, leading to the inhibited proliferation of tumor cells. Upregulation of p-eIF2α in RAW264.7 cells also inhibited expression of NFATc1 and Cat K and suppressed osteoclastogenesis, while in MC3T3 cells p-eIF2α upregulation stimulated ATF4 and promoted osteoblast differentiation. The mechanism behind p-eIF2α elevation might be linked to a kinase associated with stress to the endoplasmic reticulum, such as protein kinase RNA-like endoplasmic reticulum kinase (PERK), reported to be induced by stress to DNA synthesis in connection to Chk1 [20].

The gene expression changes identified by PCA demonstrate the potential involvement of Wnt signaling, S100A4, stress to the endoplasmic reticulum, and bone remodeling linked gene regulation in PD407824’s action. Consistent with its anti-tumor effects, PD407824 downregulated Wnt6 and Wnt7b, two genes linked to tumor progression [21, 22]. Conversely, PD407824 upregulated Wnt7a, which has been previously linked with cell growth inhibition [23]. Furthermore, a calcium-binding protein, S100A4, which is frequently upregulated in tumor cells, was downregulated by PD407824 [24]. RNA-seq analysis also identified several osteogenic genes regulated by PD407824. ATF4 is known to induce OCN expression [25]. OCN is often used as a biochemical marker for bone formation, and PD407824 was found to upregulate both OCN and ATF4 in MC3T3 cells. Finally, PD407824 was found to upregulate GADD34 and Chop. These two genes are involved in the response to stress of the endoplasmic reticulum as well as to DNA damage, and they are both implicated in apoptosis [26].

The proposed mechanism of Chk1 inhibitors’ action is illustrated (Figure 7E). Increased phosphorylation of eIF2α by Chk1 inhibitor treatment, Chk1 RNAi knockdown, and ATR inhibitor treatment, demonstrate that Chk1 inhibits eIF2α phosphorylation. Furthermore, PD407824 reduced the mRNA and protein level of S100A4 protein in 4T1.2 tumor cells. S100A4 protein level is upregulated in tumor cells, and its suppression by a neutralizing antibody is shown to reduce tumorigenesis and angiogenesis [24]. Chk1 inhibition was also shown to increase expression of LC3A/BII and cleaved caspase 3 through p-eIF2α to simulate autophagy and apoptosis, respectively, in tumor cells. Of note, autophagy is a context-dependent pro-survival pathway in response to cytotoxic stress that can lead to both tumor promotion and suppression. Autophagy in early tumorigenesis can inhibit tumor growth, but during later stages it can prolong tumor survival [27]. Taken together, these results demonstrate that Chk1 inhibition, mediated by p-eIF2α, can promote bone formation and decrease bone resorption and tumor cell growth.

While it is reported that Chk1 inhibition by knockdown or synthetic lethality by Chk1 inhibitors leads to the induction of apoptosis, the efficacy of its inhibition is dependent on the expression levels and varying mutations of p53 and p21 in tumor cells [28, 29]. Of note, p53 is mutated (Arg to Lys at 280 a.a.) in the MDA-MB-231 cell line [30] that was employed in the initial compound screening as well as in the MTT assay, while in 4T1 cells, expression of p53 is reported to be regulated by various factors, including TGF-β and c-Abl, a non-receptor protein tyrosine kinase [31]. In this study, we employed 7 tumor cell lines (3 human breast cancer cell lines and 4 mouse mammary tumor cell lines) and examined cellular proliferation. In response to 5 μM PD407824 for 3 days, the mean relative proliferation (compared to placebo) ranged from 6% to 46%. It is possible that variations in response to PD407824 among these cell lines are linked to the regulatory status of tumor suppressor genes such as p53 and p21.

Adjuvant therapy with a bisphosphonate or Denosumab is reported to help reduce the risk of bone fracture and development of bone metastasis [32, 33]. In the present study, we employed Aredia as a positive control, which, along with Zometa, is one of two FDA-approved bisphosphonates for protecting bone from metastasis [34]. In this study, no statistical difference was detected in mechanical strength of the femur between Aredia- and PD407824-treated groups. It is reported that with zoledronic acid, overall survival of breast cancer patients is not significantly improved, and no overall benefit is observed in clinical data [35, 36]. Though these therapies help protect bone, the current outcome signals a need for a more comprehensive treatment of bone metastasis.

In summary, the current study reveals differential sensitivity of three types of cells (mammary tumor, osteoblasts, and osteoclasts) to Chk1 inhibitors, and the observed difference with two selective inhibitors is beneficial for suppressing tumor growth and protecting bone. Our findings thus provide new insights into the use of Chk1 inhibitors in protecting bone from bone metastasis associated with breast cancer. Furthermore, the results herein indicate that the action of Chk1 inhibitors on bone remodeling with bone-forming osteoblasts and bone-resorbing osteoclasts is observable even in the absence of DNA damaging agents.

## MATERIALS AND METHODS

### Cell culture and agents

4T1.2 mouse mammary tumor cells [37], a non-tumorigenic epithelial cell line (CRL-3063, ATCC, Manassas, VA, USA), MDA-MB-231 with two clones (TMD and BMD) [38], and three other mammary tumor cell lines (4T1, ATCC; 67NR, Barbara Ann Karmanos Cancer Institute, Detroit, MI, USA; and E0771, CH3 BioSystems, Amherst, NY, USA) were cultured in complete DMEM, while MC3T3 osteoblast-like cells (Sigma-Aldrich, St. Louis, MO, USA) and RAW264.7 pre-osteoclast cells [39] were cultured in complete αMEM. Media was completed by supplementing with 10% fetal bovine serum (Atlanta Biologicals, GA, USA) and penicillin/streptomycin (ThermoFisher, MA, USA). Cellular proliferation was evaluated using an MTT assay (Sigma-Aldrich, St. Louis, MO, USA). PD407824 (PD) and PF477736 (PF) were employed as two selective Chk1 inhibitors, while AZ20 was used as a selective inhibitor of ATR [40]. ISRIB was used as a selective inhibitor of eIF2α phosphorylation [41], while Aredia was a positive control for inhibiting bone resorption. All agents were purchased from R&D Systems unless otherwise specified.

A wound healing scratch assay was performed to measure 2-dimensional cell motility [42]. Images of the cell-free scratch zone were obtained, and the areas newly occupied with cells were measured. An osteoclast differentiation assay was conducted by seeding RAW264.7 pre-osteoclast cells with 50 ng/ml of RANKL. The culture medium was replaced on day 4, and tartrate resistant acid phosphate (TRAP) staining was conducted on day 6 [43]. Mature osteoclasts were counted by identifying TRAP-positive multinucleated cells (>3 nuclei).

### Chk1 activity assay

To assess the inhibition of Chk1 activity by PD407824, a Chk1 Kinase Enzyme System with ADP-Glo^TM^ (Promega, Madison, WI, USA) was used, per the manufacturer’s instructions. A logarithmic dosage series of PD407824 (1 nM, 10 nM, 100 nM, 1 μM, and 10 μM) was added to the Chk1/substrate/ATP mixture and incubated for 30 min. The amount of ATP converted to ADP by Chk1 was found by using the ADP-Glo^TM^ assay system and measuring luminescence.

### qPCR and Western blot analysis

An RNeasy Plus kit (Qiagen, Germantown, MD, USA), a high capacity cDNA reverse transcription kit (Applied Biosystems, Carlsbad, CA, USA), and a Power SYBR Green PCR master mix kit (Applied Biosystems) were employed with listed PCR primers (Table 1). For detecting GFP-labeled 4T1.2 cells in the right femur, total DNA was isolated with QIAamp DNA mini kit (Qiagen) for qPCR.

**Table 1:** qPCR primers used in this study

Target	Forward primer	Backward primer
ATF4	5′- TGGCGAGTGTAAGGAGCTAGAAA -3′	5′- TCTTCCCCCTTGCCTTACG -3′
Cat K	5′- CAGCTTCCCCAAGATGTGAT -3′	5′- AGCACCAACGAGAGGAGA AA -3′
CHOP	5′- TGAGGAGAGAGAACCTGGTC -3′	5′- ACGCAGGGTCAAGAGTAGTG -3′
GADD34	5′- GAGAAGACCAAGGGACGTGG -3′	5′- AGCGAAGTGTACCTTCCGAG -3′
GFP	5′- ATCTTGAAGGGCGACGTGAG -3′	5′- TGTACACGGTGTCGAACTGG-3′
NFATc1	5′- GGTGCTGTCTGGCCATAACT -3′	5′- GCGGAAAGGTGGTATCTCAA -3′
OCN	5′- CCGGGAGCAGTGTGAGCTTA -3′	5′- AGGCGGTCTTCAAGCCATACT -3′
S100A4	5′- TCTCTCTTGGTCTGGTCTCAAC -3′	5′- TGTCACCCTCTTTGCCTGAG -3′
TRAP	5′- TCCTGGCTCAAAAAGCAGTT -3′	5′- ACATAGCCCACACCGTTCTC -3′
Wnt6	5′- GAGGGCTCCTGGAAACCTTT -3′	5′- TCCTTTAACATGGGACCACAAGT -3′
Wnt7a	5′- CCAAGGTCTTCGTGGATGCC -3′	5′- TGTTCTCCTCCAGGATCTTCCG -3′
Wnt7b	5′- CGCCATTATTTTGCACATCCT -3′	5′- CTCGAGCTCCCCACCTCTATC -3′
GAPDH	5′- TGCACCACCAACTGCTTAG -3′	5′- GGATGCAGGGATGATGTTC -3′

For Western blotting, cells were lysed by a radio-immunoprecipitation assay buffer (Santa Cruz). Proteins were fractionated by 10-15% SDS gels and electro-transferred to polyvinylidene difluoride membranes (Millipore, Billerica, MA, USA). Antibodies against ATF4, caspase 3, cathepsin K, eIF2α, p-eIF2α (Ser51), LC3A/B II, NFATc1, Chk1, p-Chk1 (Ser296) (Cell Signaling, Danvers, MA, USA), TRAP (Abcam, Cambridge, MA, USA), and β-actin (Sigma) were utilized.

### RNA-seq and principal component analysis (PCA)

A cDNA library was constructed for 4 groups (control and PD407824-treated 4T1.2 cells or MC3T3 cells) with 2 samples per group using a TruSeq Stranded mRNA Library Prep kit (Illumina, San Diego, CA, USA). Sequencing was conducted with NextSeq500 (Illumina). After quality control using FastQC (Babraham Bioinformatics, Cambridge, UK), sequenced libraries were mapped to the UCSC hg19 human genome. Principal component analysis (PCA) was performed on the log2-transformed expression data. The gene and sample locations were plotted on the first and second principal axes.

### Knockdown of Chk1 by siRNA

Cells were treated with siRNA specific to Chk1 (5′-CAA AGGACTGCTTGTCGCTG-3′; Life Technologies). A negative siRNA (Silencer Select #1, Life Technologies) was used as a nonspecific control. Cells were transiently transfected with siRNA using Lipofectamine RNAiMAX (Life Technologies) in Opti-MEM I medium [44].

### Animal models

Animal procedures were approved by the Indiana University Animal Care and Use Committee. In the mammary tumor model [45], 16 BALB/c female mice (~6 weeks, Harlan Laboratories) were utilized. 4T1.2 cells (5.0 × 10^5^ cells) were injected subcutaneously to the mammary fat pad. Daily injections of PD407824 (2 mg/kg) were administered subcutaneously. On day 18, animals were sacrificed. In the bone metastasis model [46], 20 BALB/c female mice were injected with 4T1.2 cells (1.0 × 10^5^ cells) in the right iliac artery. PD407824 was injected intraperitoneally daily at 2 mg/kg body weight, while Aredia, a bisphosphonate control, was administered weekly at 0.5 mg/kg. Animals were sacrificed on day 17.

### X-ray imaging and score

X-ray imaging was conducted using a Faxitron radiographic system (Faxitron X-ray Corporation) [47], and right femurs were scored at levels 0 to 3. Level 0 was normal with no sign of tumor; level 1 had a clear bone boundary with slight periosteum proliferation; level 2 presented clear evidence of bone damage and moderate periosteum proliferation; and level 3 showed severe bone erosion [48]. Micro-computed tomography was also performed using Skyscan 1172 (Bruker-MicroCT, Kontich Belgium) [49]. The images were reconstructed using manufacturer-provided software (nRecon v1.6.9.18).

### Mechanical testing and histology

The tibia and femur were loaded to failure by four-point bending using Electro Force 3100 (Bose, Inc.) [50]. After applying a 0.5-N preload, the bone was loaded monotonically at 0.03 mm/s. For histology, mice were injected with calcein to determine bone formation rate (BFR), mineralizing surface/bone surface (MS/BS), and mineral apposition rate (MAR) [51]. Also, H&E staining as well as tartrate-acid resistant acid phosphatase (TRAP) staining was conducted. The region ~0.8 mm proximal from the growth plate of the femur was analyzed.

### Statistical analysis

Statistical significance among groups was examined using one-way analysis of variance, and *post hoc* tests were conducted using Fisher’s protected least significant difference for pairwise comparisons. Statistical significance was assumed at *p* < 0.05. The single and double asterisks indicate *p* < 0.05 and *p* < 0.01, respectively.

## SUPPLEMENTARY MATERIALS FIGURES


