# Supplementary material for: Inhibiting checkpoint kinase 1 protects bone from bone resorption by mammary tumor in a mouse model
Source: Oncotarget. 2018 Jan 19;9(10):9364–78. doi: 10.18632/oncotarget.24286 (PMC5823640; doi:10.18632/oncotarget.24286)
Supplement: Supplementary file 1 [file oncotarget-09-9364-s001.pdf]

Inhibiting checkpoint kinase 1 protects bone from bone resorption by mammary tumor in a mouse model

SUPPLEMENTARY MATERIALS

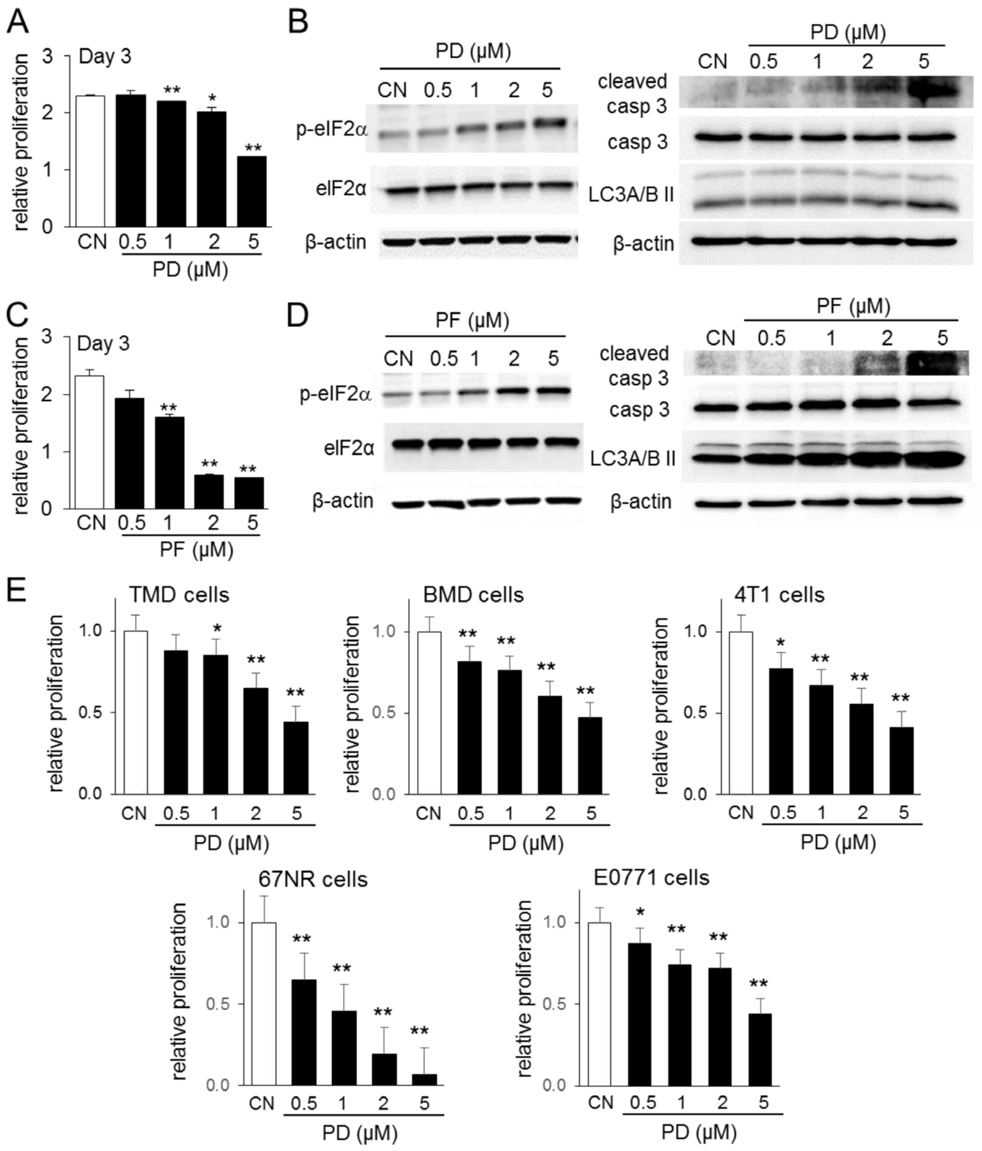

Supplementary Figure 1: Responses of breast cancer cells and mammary tumor cells to Chk1 inhibitors (PD407824 and PF477736). PD = PD407824, PF = PF477736, and CN = control. (**A**) Relative proliferation of MDA-MB-231 breast cancer cells in response to 0.5, 1, 2 or 5 µM PD in 3 days. (**B**) Dose-dependent upregulation of p-eIF2α in MDA-MB-231 cells by PD, and elevation of cleaved caspase (apoptosis marker) and LC3AB II (autophagy marker). (**C**) Relative proliferation of MDA-MB-231 cells in response to 0.5, 1, 2 or 5 µM PF in 3 days. (**D**) Dose-dependent upregulation of p-eIF2α in MDA-MB-231 breast cancer cells by PF, and elevation of cleaved caspase and LC3AB II. (**E**) Relative proliferation of two clones from MDA-MB-2231 cells (TMD and BMD cells), and three mouse mammary tumor cell lines (4T1, 67NR, and E0771) in response to 0.5, 1, 2, or 5 µM PD.

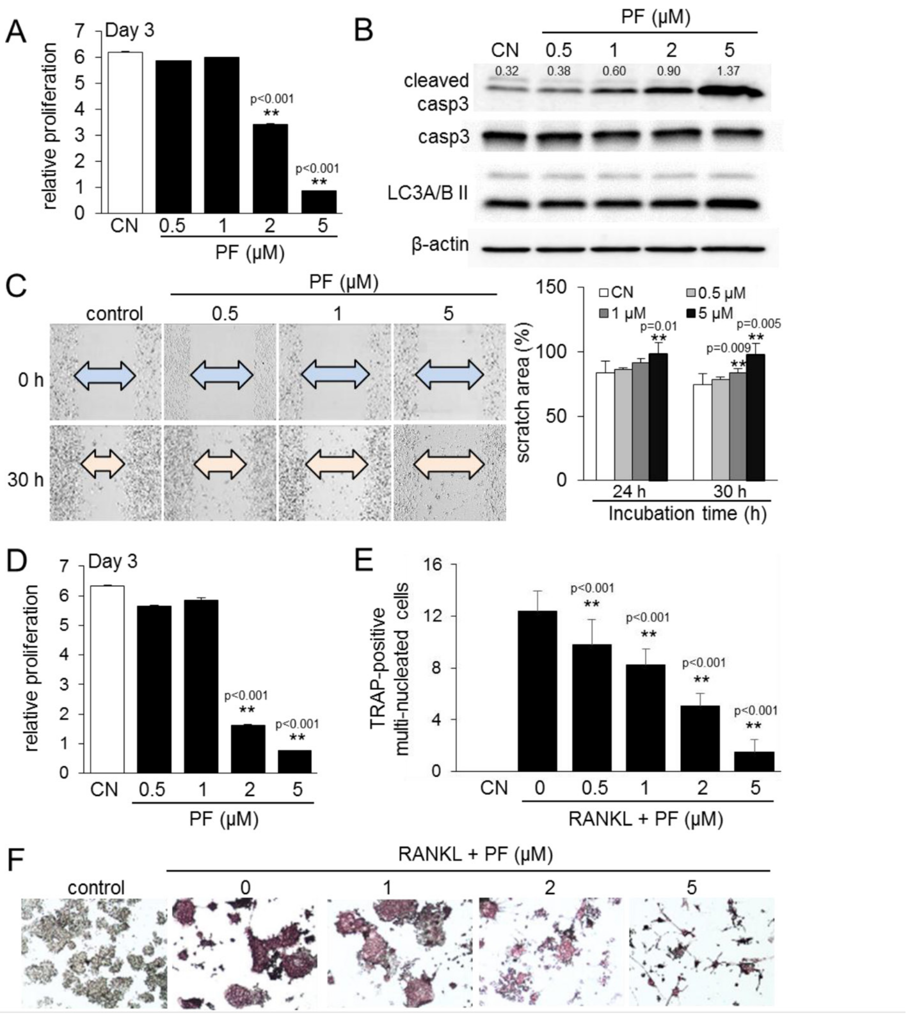

Supplementary Figure 2: Effects of PF477736 on 4T1.2 tumor cells, RAW264.7 pre-osteoclasts, and MC3T3 osteoblast-like cells. The single and double asterisks indicate $p < 0.05$ and $p < 0.01$, respectively, PF = PF477736, and CN = control. (**A**) Dose-dependent inhibition of cellular proliferation by 0.5–5 μM PF in 4T1.2 cells on day 3. (**B**) PF-driven increase in cleaved caspase 3 and LC3A/B II at 24 h in 4T1.2 cells. (**C**) Reduction in cellular motility by 0.5 – 5 μM PF in the scratch assay for 24 and 30 h in 4T1.2 cells. (**D**) Dose-dependent decrease in proliferation of RAW264.7 cells in response to 0.5 – 5 μM PF on day 3. (**E and F**) Inhibition of TRAP-positive mature osteoclast formation by 1, 2, and 5 μM PF in RAW264.7 cells on day 4 after treatment with RANKL.

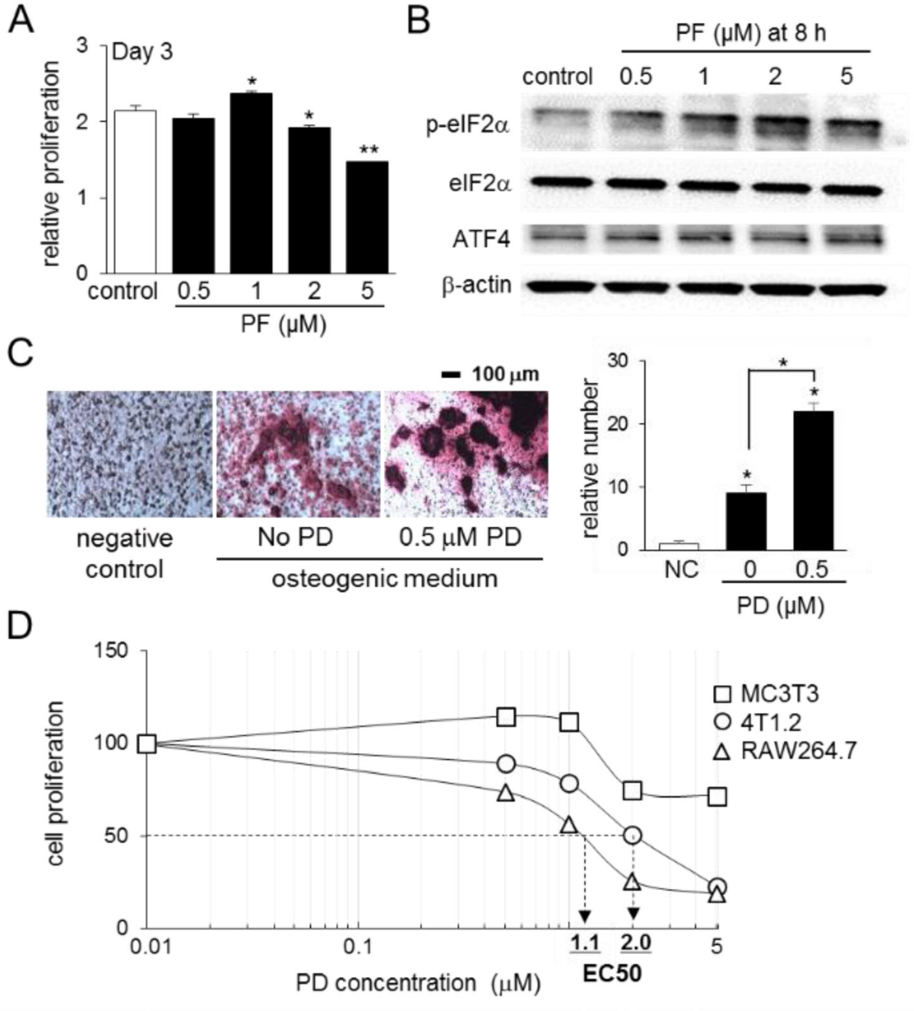

Supplementary Figure 3: Responses of MC3T3 osteoblast-like cells and EC50 (half maximal effective concentration). PF = PF477736, PD = PD407824, and CN = control. (**A**) Relative cell proliferation of MC3T3 cells in response to PF at 0.5, 1, 2, or 5 μM. (**B**) Elevation of p-eIF2α and ATF4 by PF. (**C**) Alizarin red staining and its quantification with 0.5 μM PD for 2 months in the osteogenic medium. (**D**) Estimation of EC50 of PD for 4T1.2 cells (2.0 μM) and RAW264.7 cells (1.1 μM).

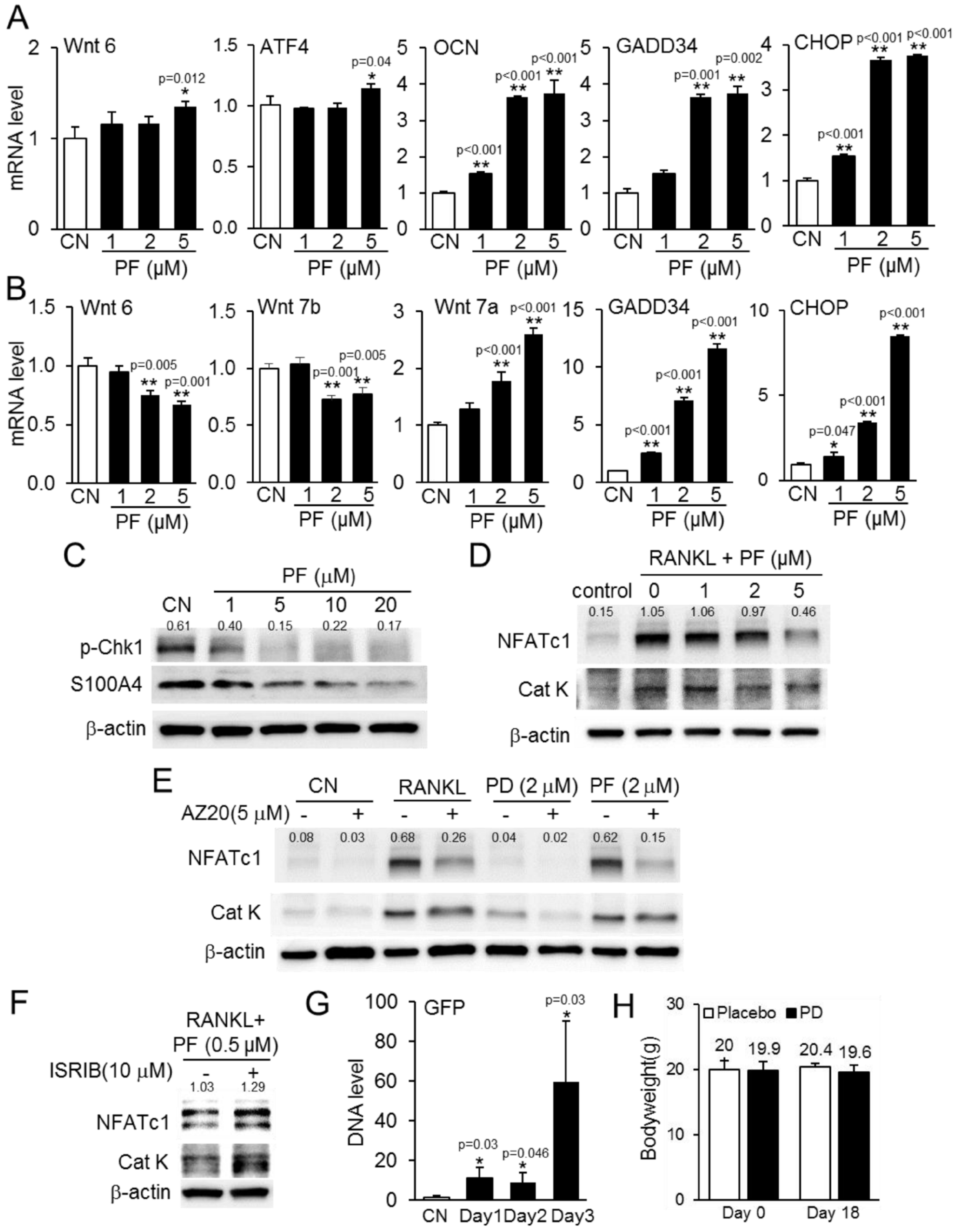

Supplementary Figure 4: Effects of PF477736, PD407824, AZ20, and ISRIB in MC3T3 cells, 4T1.2 cells, and RAW264.7 cells, as well as inoculation of GFP-labeled 4T1.2 cells in the right femur. PF = PF477736, and PD = PD407824. (**A**) Alterations in the mRNA levels of Wnt6, ATF4, OCN, GADD34, and CHOP in MC3T3 cells in response to 1 to 5 μM PF. (**B**) Alterations in the mRNA levels of Wnt6, Wnt7b, Wnt7a, GADD34, and CHOP in 4T1.2 cells in response to 1 to 5 μM PF. (**C**) Decrease in the protein levels of p-Chk1 and S100A4 in 4T1.2 cells in response to 1 to 20 μM PF. (**D**) Downregulation of NFATc1 and Cat K by 1 to 5 μM PF in RAW264.7 cells. (**E**) Reduction of NFATc1 and Cat K by 5 μM AZ20. (**F**) Suppression of PF's effect on NFATc1 and Cat K by ISRIB. (**G**) DNA level of GFP in the bone and bone marrow in the right femur on Days 1 to 3. (**H**) Body weight of mice 18 days after tumor injection in iliac artery.

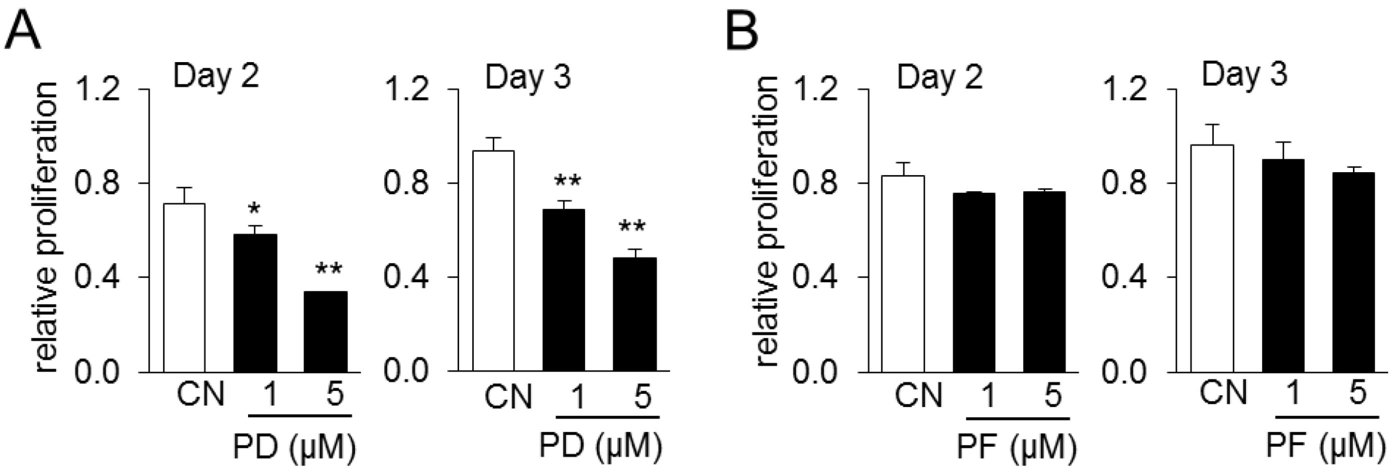

Supplementary Figure 5: Responses of non-tumorigenic mouse epithelial cell line (mouse mammary gland epithelial cells; CRL-3063). PD = PD407824, PF = PF477736, and CN = control. The single and double asterisks indicate $p < 0.05$ and $p < 0.01$, respectively. (**A and B**) Relative proliferation in 2 and 3 days in response to 1 or 5 μM PD and PF, respectively.

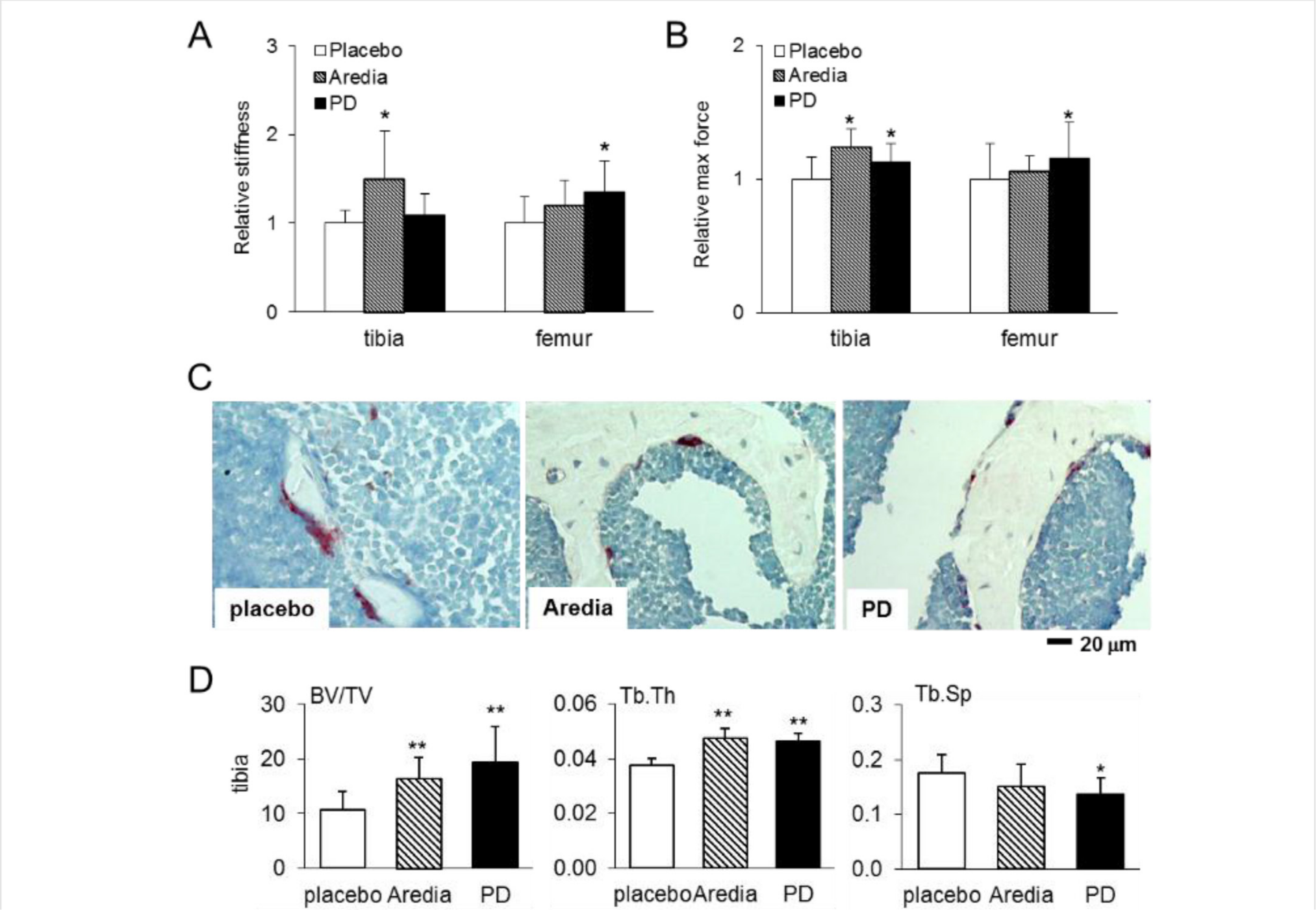

Supplementary Figure 6: Effects of daily intraperitoneal injection of 2 mg/kg PD407824 for 18 days to the mouse model of bone metastasis. The single and double asterisks indicate $p < 0.05$ and $p < 0.01$, respectively, and PD = PD407824. (**A and B**) Relative stiffness and maximum force of the tibia and femur in three groups (placebo, Aredia, and PD). The values are normalized by those for placebo. (**C**) Representative TRAP-stained sections of the distal femur in three groups (placebo, Aredia, and PD). (**D**) Three parameters for trabecular bone in the proximal tibia. Of note, BV/TV = bone volume normalized by tissue volume; Tb.Th = trabecular thickness; and Tb.Sp = trabecular separation.
